# Peripheral blood aspirates overexpressing IGF‐I *via* rAAV gene transfer undergo enhanced chondrogenic differentiation processes

**DOI:** 10.1111/jcmm.13190

**Published:** 2017-05-03

**Authors:** Janina Frisch, Patrick Orth, Ana Rey‐Rico, Jagadeesh Kumar Venkatesan, Gertrud Schmitt, Henning Madry, Dieter Kohn, Magali Cucchiarini

**Affiliations:** ^1^ Center of Experimental Orthopaedics Saarland University Medical Center Homburg/Saar Germany; ^2^ Department of Orthopaedic Surgery Saarland University Medical Center Homburg/Saar Germany

**Keywords:** cartilage repair, gene therapy, peripheral blood, rAAV vectors, IGF‐I

## Abstract

Implantation of peripheral blood aspirates induced towards chondrogenic differentiation upon genetic modification in sites of articular cartilage injury may represent a powerful strategy to enhance cartilage repair. Such a single‐step approach may be less invasive than procedures based on the use of isolated or concentrated MSCs, simplifying translational protocols in patients. In this study, we provide evidence showing the feasibility of overexpressing the mitogenic and pro‐anabolic insulin‐like growth factor I (IGF‐I) in human peripheral blood aspirates *via* rAAV‐mediated gene transfer, leading to enhanced proliferative and chondrogenic differentiation (proteoglycans, type‐II collagen, SOX9) activities in the samples relative to control (reporter rAAV‐*lacZ*) treatment over extended periods of time (at least 21 days, the longest time‐point evaluated). Interestingly, IGF‐I gene transfer also triggered hypertrophic, osteo‐ and adipogenic differentiation processes in the aspirates, suggesting that careful regulation of IGF‐I expression may be necessary to contain these events *in vivo*. Still, the current results demonstrate the potential of targeting human peripheral blood aspirates *via* therapeutic rAAV transduction as a novel, convenient tool to treat articular cartilage injuries.

## Introduction

Damaged adult hyaline articular cartilage, the tissue that is essential for weight absorption and smooth gliding of the articulating surfaces in diarthroidal joints, does not naturally undergo competent repair processes in the absence of blood vessels and lymphatic drainage that may provide regenerative progenitor cells in sites of injury 1. Lesions such as traumatic focal defects or occurring in osteoarthritis (OA) are infiltrated by cells migrating from the synovial membrane that fail to produce high‐quality repair tissue, leading to progression of the injury over time 2.

Current therapeutic options include marrow stimulation techniques to give access to the subchondral bone marrow to the lesions 2 as this compartment contains chondroreparative mesenchymal stem cells (BM‐MSCs) that may enhance the healing of the injured tissue 3, 4. Transplantation of bone marrow aspirates or concentrates has been also attempted to further enhance the processes of cartilage repair 5, 6, 7, 8, 9, 10 compared with the more tedious application of isolated and expanded MSCs 2, 11, 12, 13 or of articular chondrocytes 14, 15, leading to improved outcome in the patients. However, close evaluations revealed that the repair tissue displayed a relatively poor structural organization, integrity and biomechanical function that did not match the original hyaline cartilage. The use of peripheral blood may provide workable, less invasive translational procedures as this compartment also contains MSCs (PB‐MSCs) with the same potency for chondrogenic differentiation as that of BM‐MSCs 16, 17, 18, including *in vivo* when applied as isolated populations 9, 19, 20, 21. As such setups led again to an incomplete reconstitution of the natural cartilage structure 9, 19, 20, 21, new strategies may need to be considered to generate improved therapeutic regimens. In particular, genetic modification of such samples may be a powerful tool to stimulate the chondroreparative processes of PB‐MSCs 22. Among the various gene transfer systems available to achieve this goal, vehicles based on the human adeno‐associated virus (AAV) offer a number of benefits for translational research as recombinant AAV (rAAV) vectors are much less immunogenic and toxic and more efficient than nonviral, adenoviral and retro‐/lentiviral vectors due to the absence of viral sequences in the recombinant genome and to the long‐term maintenance of the rAAV transgenes under episomal forms 23, 24.

While genetic modification of human bone marrow aspirates *via* rAAV vectors has been reported in a variety of studies using growth (transforming growth factor β, *i.e*. TGF‐β, basic fibroblast growth factor, *i.e*. FGF‐2, IGF‐I) 4, 25, 26 and transcription factors (SOX9) 27 for chondrogenic purposes, thus far there is little evidence on the possibility of targeting human peripheral blood aspirates with this vector class. We were actually the first group to provide evidence that overexpression of TGF‐β *via* rAAV significantly enhanced the chondrogenic differentiation processes in such samples 1. Yet, as the treatment also stimulated hypertrophy and osteogenic differentiation events, new avenues of research to identify other candidate genes are needed for a safe translation of the strategy in future *in vivo* settings. In the light, the mitogenic and pro‐anabolic IGF‐I factor may be a good alternative as it displays chondroreparative activities when applied to human BM‐MSCs as isolated 28 or concentrated populations 25 as well as in articular chondrocytes 29, 30, 31. The goal of this study was thus to investigate the effects of overexpressing IGF‐I *via* rAAV upon the chondrogenic differentiation processes in human peripheral blood aspirates. This convenient approach might offer an improvement in the current therapeutic options for patients suffering from cartilage injuries while allowing for enhanced repair tissue qualities.

## Materials and methods

### Reagents

Recombinant TGF‐β (rTGF‐β) was purchased at Peprotech (Hamburg, Germany), dimethylmethyleneblue (DMMB) at Serva (Heidelberg, Germany) and diaminobenzidine (DAB) at Sigma‐Aldrich (Munich, Germany). The anti‐IGF‐I (AF‐291‐NA) antibody was from R&D Systems (Wiesbaden, Germany), the anti‐type‐II collagen (II‐II6B3) antibody from NIH Hybridoma Bank (University of Iowa, Ames, USA), the anti‐SOX9 (C‐20) antibody from Santa Cruz Biotechnology (Heidelberg, Germany), the anti‐type‐I collagen (AF‐5610) antibody from Acris (Hiddenhausen, Germany) and the anti‐type‐X collagen (COL‐10) antibody from Sigma‐Aldrich. Secondary biotinylated antibodies and the ABC kit were from Vector Laboratories (Alexis Deutschland GmbH, Grünberg, Germany). The hIGF‐I Quantikine ELISA was purchased at R&D Systems and the type‐II, ‐I and ‐X collagen ELISAs at Antibodies‐Online (Aachen, Germany).

### Peripheral blood aspirates

Peripheral blood (~3 ml) was collected in the presence of hirudin 1, 4, 25, 26, 27, 28 from donors of the Department of Orthopaedic Surgery, Saarland University Medical Center, Homburg/Saar, Germany (*n* = 4; age 42 ± 27), with informed consent. All procedures were in accordance with the Helsinki Declaration. The study was approved by the Ethics Committee of the Saarland Physicians Council (Application 39/14).

### Plasmids and rAAV vectors

All constructs applied in the study were based on pSSV9, an AAV‐2 genomic clone 32, 33. rAAV‐*lacZ* carries the *E. coli* β‐galactosidase (β‐gal) gene and rAAV‐hIGF‐I a human insulin‐like growth factor I (hIGF‐I) cDNA (536 bp) 1, 25, 28 both under the control of the cytomegalovirus immediate–early (CMV‐IE) promoter. The 293 adenovirus‐transformed embryonic kidney cell line was used to package conventional (not self‐complementary) recombinant vectors (rAAV) with helper functions provided by Adenovirus 5 and the pAd8 helper plasmid 1, 25, 28. The packaging resulted in an average of 10^10^ transgene copies/ml with 1/500 functional recombinant viral particles, as evidenced by titration *via* real‐time PCR after vector purification and dialysis 1, 25, 28.

### rAAV‐mediated gene transfer

Peripheral blood aspirates were immediately aliquoted in 96‐well plates (100 μl/well) after collection and directly mixed with the rAAV vectors using vector doses known to allow for optimal transduction efficiencies (40 μl, *i.e*. 8 × 10^5^ functional recombinant viral particles, MOI = 10 ± 3) 1. After a short incubation time (1–2 min.), 50 μl of supplement‐free DMEM was added, and the mixture was further incubated for 90 min. at 37°C and 5% CO_2_ 1. Finally, 60 μl of specific media was mixed with the transduced samples to induce chondrogenesis (4.5 g/l DMEM high glucose, 100 U/ml penicillin, 100 μl/ml streptomycin, 6.25 μg/ml insulin, 6.25 μg/ml transferrin, 6.25 μg/ml selenious acid, 5.35 μg/ml linoleic acid, 1.25 μg/ml BSA, 1 mM sodium pyruvate, 37.5 μg/ml ascorbate 2‐phosphate, 10^−7^ M dexamethasone and 10 ng/ml TGF‐β3), osteogenesis (StemPro Osteogenesis Differentiation Kit; Life Technologies, Darmstadt, Germany) or adipogenesis (StemPro Adipogenesis Differentiation Kit; Life Technologies) 1, 25. Careful medium change was performed once per week for up to 21 days of incubation 1. All aspirates formed early on clots that remained intact over the whole period of time.

### Transgene expression

Immunohistochemical staining was performed to detect transgene (IGF‐I) expression using a specific primary antibody, a biotinylated secondary antibody and the ABC method using DAB as the chromogen 1. Sections were recorded under light microscopy (Olympus BC45; Olympus, Hamburg, Germany) at 20× magnification for histomorphometric analyses 1. IGF‐I secretion was monitored by ELISA using cell culture supernatants at the denoted time‐points and 24 hrs after medium change to conditioned medium 1 with a GENios spectrophotometer (Tecan, Crailsheim, Germany).

### Biochemical analyses

Aspirates were collected in a volume of 100 μl of fresh, supplement‐free DMEM, followed by papain digestion (final concentration: 75 μg/ml; 60 min. at 60°C) 1. The amounts of total proteins were detected using the Pierce BCA Protein Assay Kit (Pierce, ThermoFisher Scientific, Schwerte, Germany) for normalization of biochemical measurements 1. Evaluation of the DNA content was carried out using the Hoechst 22358 assay and the proteoglycan contents by binding to the DMMB dye 1. Type‐II, ‐I and ‐X collagen contents were monitored by respective ELISA according to the manufacturer's protocols 1. Osteogenesis was assessed by mixing osteogenically induced aspirates with an equal volume of specific substrate buffer (4‐nitrophenyl phosphate—pNPP—mixed 1:1 with 4.8% 2‐amino‐2‐methyl‐1‐propanol—2‐AMP) to analyse ALP activity by absorbance measurements 25. Adipogenesis was evaluated *via* Oil Red O staining as previously described 25. Briefly, adipogenically induced aspirates were mixed with 150 μl of staining solution (three volumes of Oil Red O 0.3% in 2‐propanol and two volumes of H_2_O), followed by incubation for 15 min. at room temperature and final dissolution in 100% 2‐propanol for absorbance measurements. Evaluations were performed on a GENios spectrophotometer/fluorometer (Tecan).

### Histological and immunohistochemical analyses

The samples were fixed in 4% formalin and embedded in paraffin after dehydration in graded alcohols, followed by sectioning at 3 μm to be placed on histological slides 1. Haematoxylin eosin staining (H&E) was carried out to evaluate cellularity 1. The deposition of proteoglycans and matrix mineralization was visualized *via* toluidine blue and alizarin red staining respectively 1. Immunohistochemical analyses were performed to detect the expression of type‐II, ‐I and ‐X collagen as well as SOX9 using specific primary antibodies, biotinylated secondary antibodies and the ABC method with DAB as the chromogen 1. Stained sections were recorded under light microscopy (Olympus BX45) at 20× magnification for histomorphometric analyses 1.

### Histomorphometry

Staining intensities on histological (H&E, toluidine blue, alizarin red) and immunohistochemical (IGF‐I, type‐II, ‐I and ‐X collagen, SOX9) sections were analysed by measurement of pixels per standardized area (pixels/mm²) 1. Images at 20× magnification were converted to greyscale mode, and inverted, background signal was adapted for comparable range and the mean grey value per total area covered with cells was detected 1. All data were collected at three random standardized sites or using 10 serial histological and immunohistochemical sections for each parameter, test and replicate condition using the CellSens Standard programme (Olympus) and Adobe Photoshop (Adobe Systems, Unterschleissheim, Germany) 1.

### Real‐time RT‐PCR analyses

Aspirates collected in a total volume of 100 μl fresh, supplement‐free medium, were mixed with 6 volumes of TRIzol reagent (Ambion^®^ Life Technologies) and 3 volumes of chloroform, followed by density gradient centrifugation 1. The nucleic acid containing clear phase was further used to perform RNA isolation with the RNeasy Protect Mini Kit (Qiagen, Hilden, Germany), including an on‐column RNase‐free DNase treatment (Qiagen) and elution in 30 μl of RNase‐free water 1. Small aliquots (8 μl) were used for reverse transcription using the 1st Strand cDNA Synthesis kit for RT‐PCR (AMV; Roche Applied Science), followed by amplification *via* real‐time RT‐PCR with 2 μl of the newly synthesized cDNA using the Brilliant SYBR Green QPCR Master Mix (Stratagene, Agilent Technologies, Waldbronn, Germany) and a Mx3000P QPCR operator system (Stratagene) 1. The following cycle conditions were used: (95°C, 10 min.), amplification by 55 cycles (denaturation at 95°C, 30 sec.; annealing at 55°C, 1 min.; extension at 72°C, 30 sec.), denaturation (95°C, 1 min.) and final incubation (55°C, 30 sec.). Primers were purchased at Invitrogen (Darmstadt, Germany) applied at a final concentration of 150 nm as follows: aggrecan (ACAN) (chondrogenic marker) (forward 5′‐GAGATGGAGGGTGAGGTC‐3′; reverse 5′‐ACGCTGCCTCG GGCTTC‐3′), type‐II collagen (COL2A1) (chondrogenic marker) (forward 5′‐GGACTTTTCTCCCCTCTCT‐3′; reverse 5′‐GACCCGAAGGTC TTACAGGA‐3′), SOX9 (chondrogenic marker) (forward 5′‐ACACACAGCT CACTCGACCTTG‐3′; reverse 5′‐GGGAATTCTGGTTGGTCCTCT‐3′), type‐I collagen (COL1A1) (osteogenic marker) (forward 5′‐ACGTCCTGGTGAA GTTGGTC‐3′; reverse 5′‐ACCAGGGAAGCCTCTCTCTC‐3′), type‐X collagen (COL10A1) (marker of hypertrophy) (forward 5′‐CCCTCTTGTTAGTGCCAACC‐3′; reverse 5′‐AGATTCCAGTCCTTGGGTCA‐3′), alkaline phosphatase (ALP) (osteogenic marker) (forward 5′‐TGGAGCTTCAGAAGCT CAACACCA‐3′; reverse 5′‐ATCTCGTTGTCTGAG TACCAGTCC‐3′), matrix metalloproteinase 13 (MMP13) (marker of terminal differentiation) (forward 5′‐AATTTTCACTTTTGGCAATGA‐3′; reverse 5′‐CAAATAATTTATGAA AAAGGGATGC‐3′), runt‐related transcription factor 2 (RUNX2) (osteogenic marker) (forward 5′‐GCAGTTCCCAAGCATTTCAT‐3′; reverse 5′‐CACTCTGGCTTTGGGA AGAG‐3′) and glyceraldehyde‐3‐phosphate dehydrogenase (GAPDH) (housekeeping gene and internal control) (forward 5′‐GAAGGTGAAGGTCGGAGTC‐3′; reverse 5′‐GAAGATGGTGATGGGATT TC‐3′) 1. Threshold cycle (Ct) values were measured on the MxPro QPCR Software (Stratagene), and gene expression profiles were evaluated after normalization to GAPDH using the 2^−ΔΔCt^ method 1.

### Statistical analysis

Two independent experiments were performed for each patient and condition to generate duplicates. All samples were tested for each evaluation. Data are represented as mean ± standard deviation (S.D.), and the *t*‐test with normal distribution of data was used where appropriate, considering *P* ≤ 0.05 for statistically significant.

## Results

### Successful rAAV‐mediated gene transfer and overexpression of IGF‐I in chondrogenically induced human peripheral blood aspirates

Human peripheral blood aspirates were first transduced with the candidate rAAV‐hIGF‐I vector in order to examine the possibility of overexpressing the growth factor in the samples over time in conditions of continuous chondrogenic induction relative to control (no vector treatment, rAAV‐*lacZ* gene transfer) conditions.

**Figure 1:**
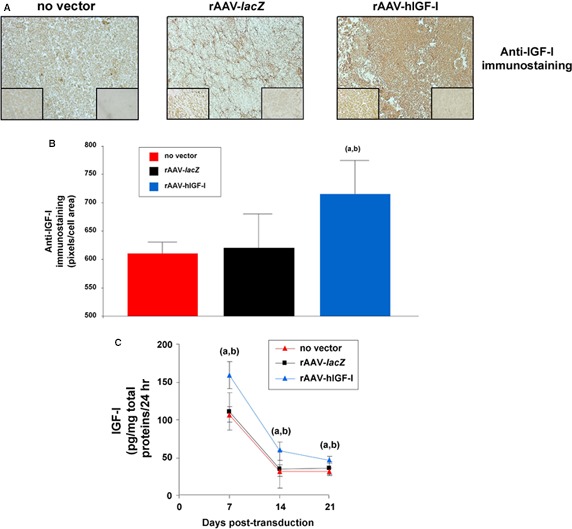
Transgene (IGF‐I) expression analyses in rAAV‐modified, chondrogenically induced human peripheral blood samples. Aspirates (*n* = 4) were transduced with rAAV‐*lacZ* or rAAV‐hIGF‐I (40 μl each vector) or let untreated and kept for up to 21 days in chondrogenic medium as described in the Materials and methods and analysed to monitor IGF‐I transgene expression *via* immunohistochemistry (**A**) (magnification x20; left insets at magnification x40; right inserts at magnification ×40 show sections without primary antibody; all representative data) with corresponding histomorphometric analyses (**B**) and by ELISA (**C**). Statistically significant compared with ^a^no vector treatment and ^b^rAAV‐*lacZ*.

An immunohistochemical analysis revealed significantly higher IGF‐I staining intensities in aspirates transduced with rAAV‐hIGF‐I compared with the control conditions (up to 1.2‐fold difference on day 21, *P* ≤ 0.05; Fig. 1A and B). An evaluation of the IGF‐I production levels in the samples by ELISA further showed significantly higher amounts of growth factor expression with rAAV‐hIGF‐I relative to the control conditions at any time‐point of the analysis (up to 1.7‐fold difference, *P* ≤ 0.05), while no differences were noted between rAAV‐*lacZ* and a lack of vector treatment (*P* ≥ 0.050; Fig. 1C).

### Enhanced proliferative and chondrogenic activities in chondrogenically induced human peripheral blood aspirates upon rAAV‐mediated IGF‐I overexpression'

Human peripheral blood aspirates were then transduced with rAAV‐hIGF‐I to test the potential effects of IGF‐I overexpression on the proliferative and chondrogenic activities in the samples under continuous chondrogenic induction *versus* control (rAAV‐*lacZ*) transduction. As we previously reported that rAAV‐mediated gene transfer has no deleterious effects on the multipotency of such aspirates 1 and in the light of our results above on transgene expression showing no statistically significant difference between the two control groups, the condition where the vectors were omitted was not included here.

**Figure 2:**
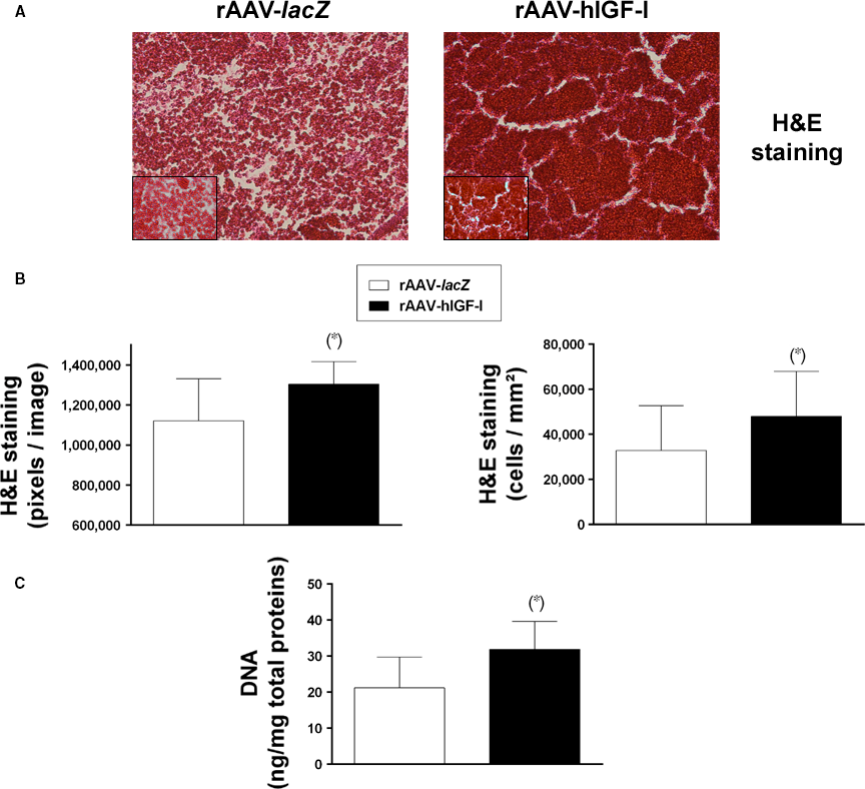
Proliferative activities in rAAV‐modified, chondrogenically induced human peripheral blood aspirates. Aspirates (*n* = 4) were transduced as described in Fig. 1 and processed after 21 days for H&E staining (**A**) (magnification x20; insets at magnification x40; all representative data) with corresponding histomorphometric analyses (**B**) and to measure the DNA contents (**C**) as described in the Materials and methods. *Statistically significant compared with rAAV‐*lacZ*.

Significantly increased cellularity was observed when applying rAAV‐hIGF‐I to the aspirates compared with rAAV‐*lacZ* as noted on H&E‐stained histological sections (up to 1.1‐fold difference on day 21, *P* ≤ 0.05; Fig. 2A and B). Similar results were noted when analysing the DNA contents in the transduced aspirates (1.5‐fold increases with rAAV‐hIGF‐I relative to rAAV‐*lacZ* on day 21, *P* ≤ 0.05; Fig. 2C).

**Figure 3:**
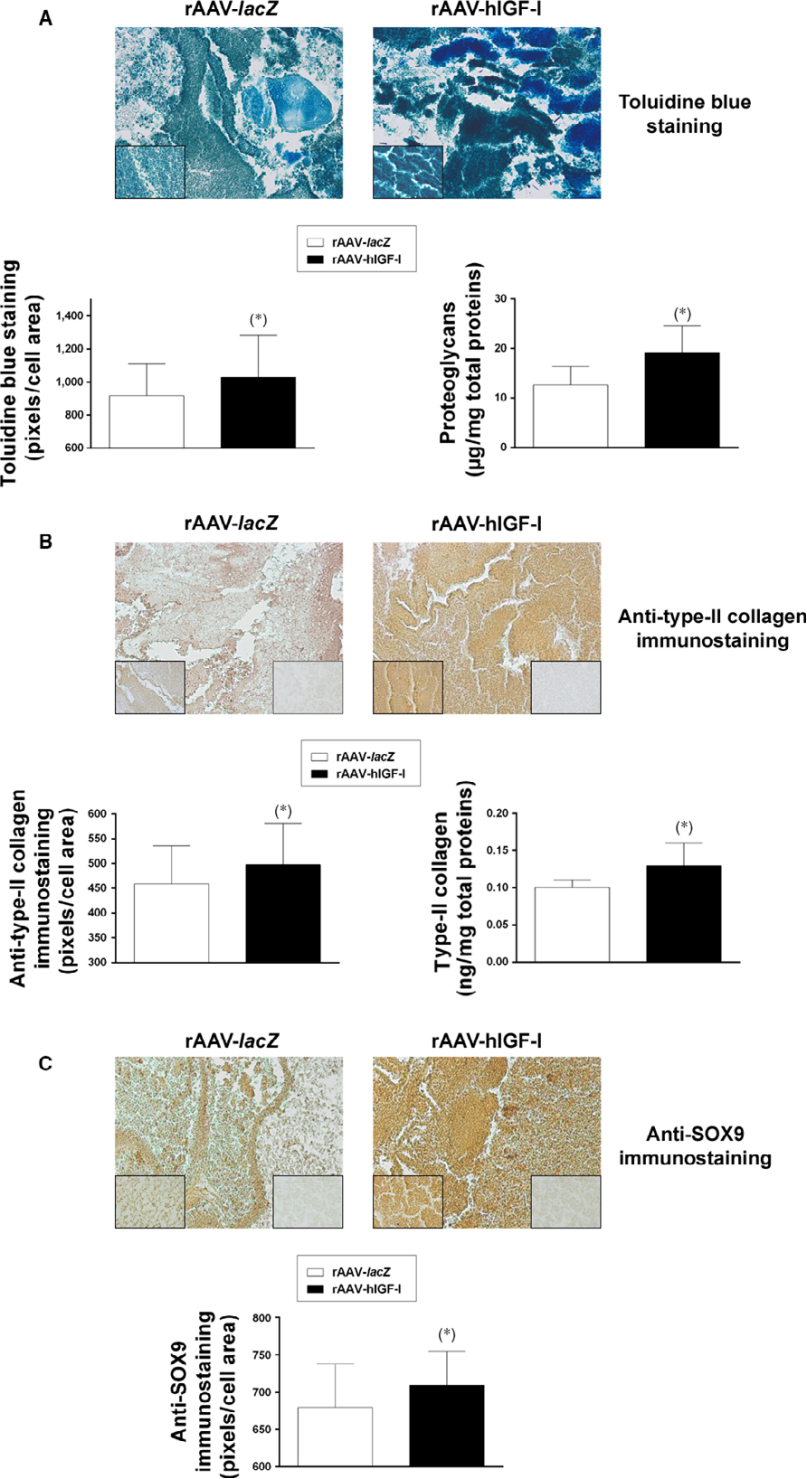
Chondrogenic marker expression in rAAV‐modified, chondrogenically induced human peripheral blood samples. Aspirates (*n* = 4) were transduced as described in Fig. 1 and processed after 21 days for toluidine blue staining with histomorphometric analyses and to measure the proteoglycan contents (**A**) (magnification x20; insets at magnification x40; all representative data) for type‐II collagen immunostaining with histomorphometric analyses and to measure the type‐II collagen contents (**B**) (magnification x20; left insets at magnification x40; right inserts at magnification ×40 show sections without primary antibody; all representative data) and for SOX9 immunostaining with histomorphometric analyses (**C**) (magnification x20; left insets at magnification x40; right inserts at magnification ×40 show sections without primary antibody; all representative data) as described in the Materials and methods. *Statistically significant compared with rAAV‐*lacZ*.

**Figure 4:**
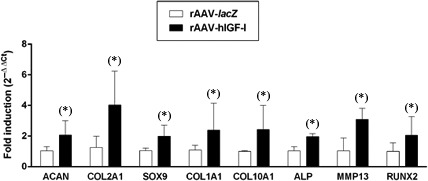
Real‐time RT‐PCR analyses in rAAV‐modified, chondrogenically induced human peripheral blood aspirates. Aspirates (*n* = 4) were transduced as described in Fig. 1 and processed after 21 days to analyse the gene expression profiles of aggrecan (ACAN), type‐II collagen (COL2A1), the transcription factor SOX9, type‐I collagen (COL1A1), type‐X collagen (COL10A1), alkaline phosphatase (ALP), matrix metalloproteinase 13 (MMP13) and of the transcription factor RUNX2, with GAPDH serving as a housekeeping gene and internal control for normalization (primers listed in the Materials and methods section). Fold inductions were measured for each target using the 2^−ΔΔCt^ method relative to rAAV‐*lacZ*‐treated aspirates. *Statistically significant compared with rAAV‐*lacZ*.

Enhanced levels of chondrogenic marker expression were also achieved in the aspirates upon overexpression of IGF‐I compared with control (*lacZ*) treatment as seen by significantly increased staining and immunostaining intensities of toluidine blue, type‐II collagen and SOX9 (always 1.1‐fold difference on day 21, *P* ≤ 0.05; Fig. 3A–C), a finding confirmed when estimating the proteoglycan and type‐II collagen contents in the samples (up to 1.5‐fold difference on day 21, *P* ≤ 0.05; Fig. 3A and B). Furthermore, a real‐time RT‐PCR analysis revealed higher gene expression profiles for aggrecan (ACAN), COL2A1 and SOX9 in rAAV‐hIGF‐I‐treated aspirates *versus* rAAV‐*lacZ* (2.0‐, 3.2‐ and 1.9‐fold difference on day 21 respectively, *P* ≤ 0.05; Fig. 4).

### Effects of rAAV‐mediated IGF‐I overexpression on the hypertrophic and terminal differentiation activities in chondrogenically induced human peripheral blood aspirates

Human peripheral blood aspirates were next transduced with rAAV‐hIGF‐I to test the potential effects of IGF‐I overexpression on the hypertrophic and terminal differentiation activities in the samples under continuous chondrogenic induction *versus* control (rAAV‐*lacZ*) transduction.

**Figure 5:**
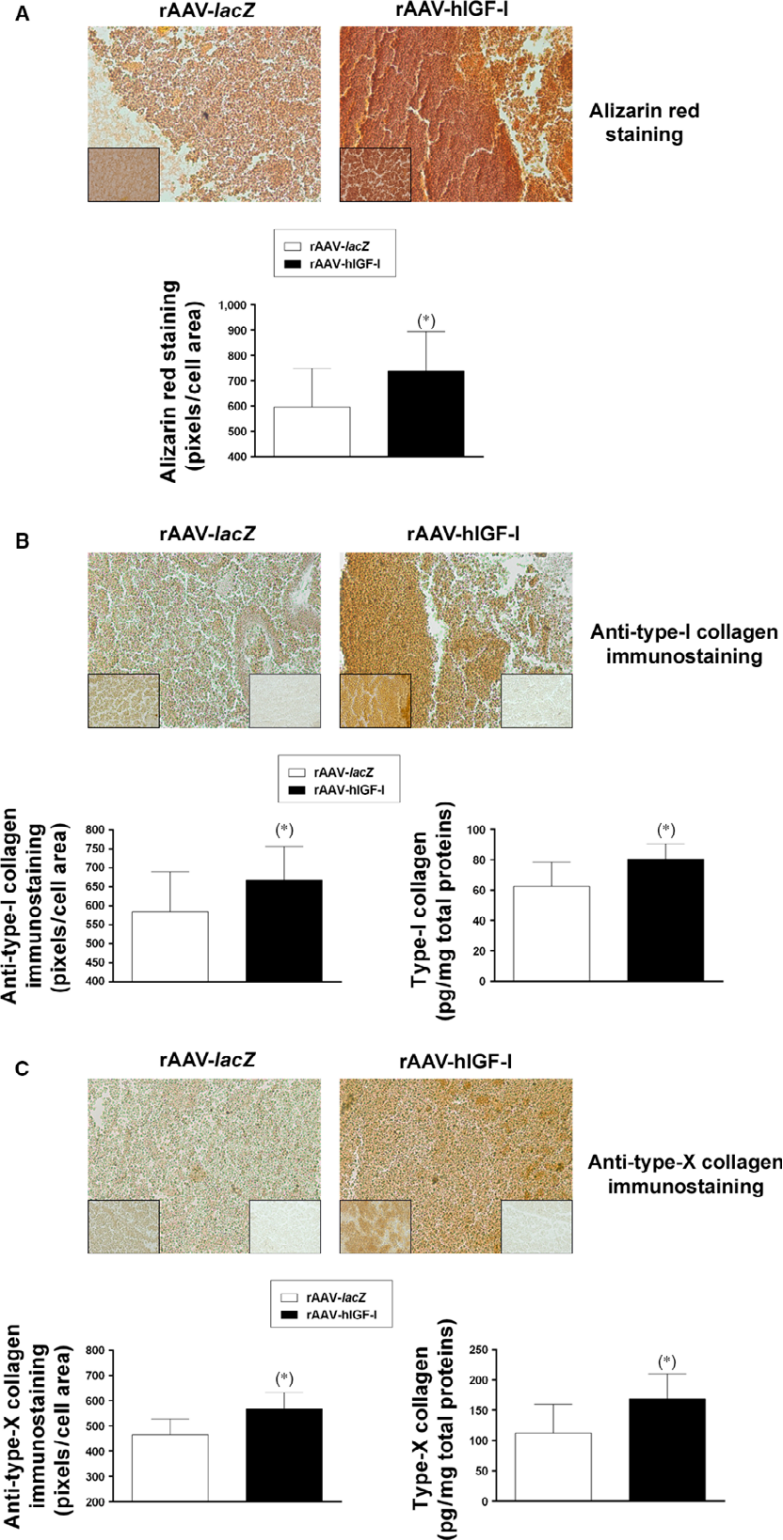
Hypertrophic and osteogenic differentiation in rAAV‐modified, chondrogenically induced human peripheral blood aspirates. Aspirates (*n* = 4) were transduced as described in Fig. 1 and processed after 21 days for alizarin red staining with histomorphometric analyses (**A**) (magnification x20; all representative data), for type‐I collagen immunostaining with histomorphometric analyses and to measure the type‐I collagen contents (**B**) (magnification x20; left insets at magnification x40; right inserts at magnification ×40 show sections without primary antibody; all representative data) and for type‐X collagen immunostaining with histomorphometric analyses and to measure the type‐X collagen contents (**C**) (magnification x20; left insets at magnification x40; right inserts at magnification ×40 show sections without primary antibody; all representative data) as described in the Materials and methods. *Statistically significant compared with rAAV‐*lacZ*.

Enhanced levels of matrix calcification were achieved in the aspirates upon overexpression of IGF‐I compared with control (*lacZ*) treatment as seen by significantly increased alizarin red staining (1.2‐fold difference on day 21, *P* ≤ 0.05; Fig. 5A). Significantly increased expression of type‐I and ‐X collagen was also noted by immunohistochemical analyses in the presence of rAAV‐hIGF‐I relative to rAAV‐*lacZ* (up to 1.2‐fold difference on day 21, *P* ≤ 0.05; Fig. 5B and5C), a finding corroborated by an estimation of the type‐I and ‐X collagen contents in the samples (up to 1.5‐fold difference on day 21, *P* ≤ 0.05; Fig. 5B and C). A real‐time RT‐PCR analysis finally demonstrated higher gene expression profiles for COL1A1, COL10A1 and for the osteogenic marker alkaline phosphatase (ALP) in rAAV‐hIGF‐I‐treated aspirates *versus* rAAV‐*lacZ* (2.2‐, 2.4‐ and 1.9‐fold difference on day 21, respectively, *P* ≤ 0.05; Fig. 4), concomitantly with increased expression of the marker of terminal differentiation MMP13 and of the osteogenic transcription factor RUNX2 (3.0‐ and 2.1‐fold difference on day 21, respectively, *P* ≤ 0.05; Fig. 4).

### Effects of rAAV‐mediated IGF‐I overexpression on the osteogenic and adipogenic differentiation processes in osteogenically and adipogenically induced human peripheral blood aspirates

Human peripheral blood aspirates were finally transduced with rAAV‐hIGF‐I *versus* rAAV‐*lacZ* to test the potential effects of IGF‐I overexpression on the osteogenic and adipogenic differentiation processes in the samples under continuous osteogenic or adipogenic induction.

**Figure 6:**
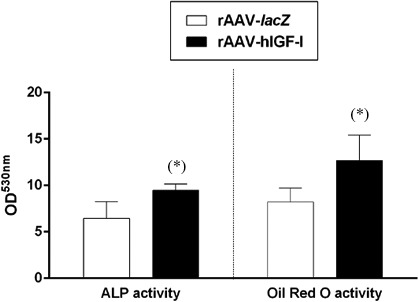
Osteogenic and adipogenic differentiation processes in rAAV‐modified, osteogenically and adipogenically induced human peripheral blood aspirates. Aspirates (*n* = 4) were transduced as described in Figure 1, and the osteogenic and adipogenic differentiation pathways were assessed after 21 days by analysis of the ALP and Oil Red O activities, respectively as described in the Materials and methods. *Statistically significant compared with rAAV‐*lacZ*.

Application of the IGF‐I vector led to higher osteogenic and adipogenic activities in the aspirates compared with control (*lacZ*) treatment as noted by an estimation of the ALP and Oil Red O activities respectively (always 1.5‐fold difference on day 21, *P* ≤ 0.05; Fig. 6).

## Discussion

Direct modification of peripheral blood aspirates using the clinically relevant, highly effective rAAV gene vectors is a promising strategy to enhance the chondrogenic differentiation of PB‐MSCs for further convenient therapeutic application in articular cartilage lesions 1. In the light of our previous work showing the chondroreparative potency of rAAV IGF‐I gene transfer in concentrated BM‐MSCs 25, the approach developed here was to test whether this active construct was capable of enhancing the chondrogenic activities in freshly isolated peripheral blood aspirates from patients as the ultimate goal for single‐step, minimal invasive therapy of cartilage injury.

The data first indicate that effective, significant overexpression of IGF‐I was achieved in human peripheral blood aspirates upon rAAV gene transfer relative to the control (*lacZ*) treatment for up to 21 days, the longest time‐point evaluated, concordant with our findings in human bone marrow concentrates 25. Interestingly, while early on the levels of IGF‐I production were higher in the IGF‐I‐treated peripheral blood aspirates (~150 *versus* 97 pg/mg total proteins/24 hrs in bone marrow samples, *i.e*. an ~1.5‐fold difference, *P* ≤ 0.05), they decreased over time and became inferior to those noted in the bone marrow (~47 *versus* 106 pg/mg total proteins/24 hrs, *i.e*. an ~2.3‐fold difference, *P* ≤ 0.05) 25. This might be due to dissimilar biochemical (paracrine factors) and cellular microenvironments in the samples (different representation of MSCs: ~0.0002% MSCs in the blood *versus* ~1% in the marrow; distinct populations of fibroblasts and haematopoietic cells) 16, 17, 18, 34, 35, 36, 37, 38, to a variability of permissivity to rAAV gene transfer and intracellular transgene processing in the samples 39 and/or to different clonal expansion of IGF‐I‐transduced populations in the aspirates 1, 25. Work is ongoing to appreciate such discrepancies between samples by comparing patient‐matched blood and marrow concentrates and to characterize the cell subpopulations amenable to rAAV gene transfer in chondrogenically induced the peripheral blood aspirates by immunophenotyping 1. In the light of our previous findings in bone marrow aspirates 25, it is probable that in conditions of continuous chondrogenic induction, mostly MSCs capable of competently committing towards the chondrocyte phenotype in such an environment 34, 38 may contribute to the chondrogenic differentiation processes.

We next show that rAAV‐mediated IGF‐I overexpression enhanced the proliferative and chondrogenic activities in the peripheral blood aspirates over time (21 days) and relative to the control treatment, again in good agreement with our findings in human bone marrow concentrates 25 and with the properties of the growth factor 40. Of note, the proliferative indices in the IGF‐I‐treated peripheral blood aspirates were lower than those achieved in the bone marrow (~32 *versus* 2700 ng DNA/mg total proteins, *i.e*. an ~84‐fold difference, *P* ≤ 0.05) 25, possibly due to the differences in IGF‐I production levels over time between the samples, to distinct levels of IGF‐I receptor membrane expression in cells forming the aspirates, and/or to the lower frequency of chondrogenically competent MSCs in the blood relative to the bone marrow that may mostly be the subpopulation undergoing cell division over time in conditions of continuous chondrogenic stimulation 1. Furthermore, the levels of chondrogenic activities in the IGF‐I‐treated peripheral blood aspirates were also inferior to those noted in the bone marrow (~20 *versus* 40 μg proteoglycans/mg total proteins and ~0.13 *versus* 0.30 ng type‐II collagen/mg total proteins, *i.e*. an ~2‐ to 2.3‐fold difference, *P* ≤ 0.05) 25, again possibly reflecting the lower levels of IGF‐I produced *via* rAAV in the blood samples, the lesser representation of MSCs in these samples that may mostly be prone to chondrogenic commitment under continuous specific stimulation 1 and/or to discrepancies in the state of activation of these cells over time in the blood aspirates in their particular microenvironment. For comparison, similar levels of proliferation and chondrogenic activities were reported when applying a rAAV TGF‐β construct to human peripheral blood aspirates using identical conditions of chondrogenic induction 1.

We finally demonstrate that gene transfer with rAAV IGF‐I also promoted hypertrophic and osteogenic differentiation in peripheral blood aspirates over time and relative to the control treatment, corroborating our findings in human bone marrow concentrates 25 and with the properties of the growth factor 41. Strikingly, the extent of such events was lower in the IGF‐I‐treated peripheral blood aspirates compared with the bone marrow (~80 *versus* 300 pg type‐I collagen/mg total proteins and ~170 *versus* 400 pg type‐X collagen/mg total proteins, *i.e*. an ~2.4‐ to 3.8‐fold difference, *P* ≤ 0.05) 25, again possibly resulting from lower levels of IGF‐I production *via* rAAV in the blood or to a more favourable microenvironment in this compartment that may allow to better contain such undesirable processes. Of further note, the levels of hypertrophic and osteogenic differentiation achieved in the human peripheral blood aspirates here with rAAV IGF‐I were inferior to those seen when using an rAAV TGF‐β construct in similar conditions of chondrogenic induction (~170 *versus* 2600 pg type‐X collagen/mg total proteins, *i.e*. an ~15.3‐fold difference, *P* ≤ 0.05) 1.

In summary, the present study shows the value of modifying peripheral blood aspirates *via* rAAV gene transfer as a means to enhance chondrogenic processes in the view of future implantation protocols to conveniently treat articular cartilage lesions. As hypertrophy and osteogenic differentiation were also induced by rAAV‐hIGF‐I here, regulation of transgene expression may need attention prior to translation in clinically relevant, orthotopic models of cartilage injury *in vivo* 42. This might be achieved by employing tissue‐specific (*SOX9*, type‐II collagen) or regulatable (tetracycline‐sensitive) promoters or by co‐applying vectors coding for antihypertrophic factors 43 like FGF‐2 44, the family of SOX transcription factors 27, 45, 46, or an anti‐Cbfa‐1 siRNA 47. However, no clear evidence of hypertrophy and terminal differentiation could be documented when providing the current rAAV IGF‐I vector to experimental osteochondral lesions in the knee joints of rabbits 48. Overall, the use of such manipulated blood samples may find value to allow for less invasive and less cumbersome treatments for the affected patients, avoiding the need for cell isolation, expansion and re‐implantation in sites of cartilage injury.

## Conflict of interest

The authors confirm that there are no conflict of interests.
